# Patient-Derived Papillary Thyroid Cancer Organoids for Radioactive Iodine Refractory Screening

**DOI:** 10.3390/cancers12113212

**Published:** 2020-10-31

**Authors:** Luc H.J. Sondorp, Vivian M.L. Ogundipe, Andries H. Groen, Wendy Kelder, Annelies Kemper, Thera P. Links, Robert P. Coppes, Schelto Kruijff

**Affiliations:** 1 Department of Surgical Oncology, University of Groningen, University Medical Center Groningen, 9713 GZ Groningen, The Netherlands; l.h.j.sondorp@umcg.nl (L.H.J.S.); a.h.groen-4@umcutrecht.nl (A.H.G.); 2 Department of Biomedical Sciences of Cell & Systems–Section Molecular Cell Biology, University of Groningen, University Medical Center Groningen, 9700 RB Groningen, The Netherlands; v.m.l.ogundipe@umcg.nl; 3 Department of Radiation Oncology, University of Groningen, University Medical Center Groningen, 9700 RB Groningen, The Netherlands; 4 Department of Surgery, Martini Hospital, 9728 NT Groningen, The Netherlands; w.kelder@mzh.nl; 5 Department of Surgery, Treant Hospital, 7909 AA Hoogeveen, The Netherlands; a.kemper@treant.nl; 6 Department of Endocrinology, University of Groningen, University Medical Center Groningen, 9700 RB Groningen, The Netherlands; t.p.links@umcg.nl

**Keywords:** patient-derived tumor organoids, three-dimensional culture, tumor organoids, papillary thyroid carcinoma, cancer stem cells, treatment prediction, RAI therapy

## Abstract

**Simple Summary:**

Over the past three decades, the incidence of thyroid cancer has been rising, with 90% being the well-differentiated thyroid cancer subtype. After diagnosis and surgical removal of the thyroid gland, radioactive iodine is administered to induce a localized post-operative radiation treatment. However, in 15-33% of papillary thyroid cancer cases, the cells are unable to take up radioactive iodine, resulting in an ineffective treatment which sometimes has severe side effects. Pre-treatment diagnosis of non-responding patients would prevent ineffective and toxic iodine treatment. Therefore, in this study, we developed a patient-derived papillary thyroid cancer organoid model. Patient-derived organoids responding or not responding to radioactive iodine clearly resembled the tumor of origin, but showed clear differences in sodium/iodide symporter expression. Our results indicate that thyroid cancer organoids might be a suitable tool for the early diagnosis of non-responding patients, in order to eventually reduce radioactive iodine overtreatment and its many side effects for thyroid cancer patients.

**Abstract:**

Patients with well-differentiated thyroid cancer, especially papillary thyroid cancer (PTC), are treated with surgical resection of the thyroid gland. This is followed by post-operative radioactive iodine (I^131^), resulting in total thyroid ablation. Unfortunately, about 15-33% of PTC patients are unable to take up I^131^, limiting further treatment options. The aim of our study was to develop a cancer organoid model with the potential for pre-treatment diagnosis of these I^131^-resistant patients. PTC tissue from thirteen patients was used to establish a long-term organoid model. These organoids showed a self-renewal potential for at least five passages, suggesting the presence of cancer stem cells. We demonstrated that thyroid specific markers, a PTC marker, and transporters/receptors necessary for iodine uptake and thyroid hormone production were expressed on a gene and protein level. Additionally, we cultured organoids from I^131^-resistant PTC material from three patients. When comparing PTC organoids to radioactive iodine (RAI)-refractory disease (RAIRD) organoids, a substantial discordance on both a protein and gene expression level was observed, indicating a treatment prediction potential. We showed that patient-derived PTC organoids recapitulate PTC tissue and a RAIRD phenotype. Patient-specific PTC organoids may enable the early identification of I^131^-resistant patients, in order to reduce RAI overtreatment and its many side effects for thyroid cancer patients.

## 1. Introduction

Over the past three decades, an increase in the incidence of thyroid cancers has been observed, with little to no change in mortality [1,2]. If recent trends are maintained, the prediction is that thyroid cancer will be in the top five most common cancers by the year 2030 [3,4]. The rise of thyroid cancer incidence is probably the result of increased diagnostic modalities, changing demographics, and environmental risk factors, resulting in more thyroid cancer incidentalomas [1,5,6,7].

Thyroid cancer can be divided on the basis of histology into well-differentiated, poorly differentiated, and undifferentiated thyroid cancer [6]. Well-differentiated thyroid cancer (WDTC) is the most prevalent subtype of thyroid cancer, representing 90% of cases. WDTC can be subdivided into papillary thyroid cancers (PTCs, 75–85% of cases), follicular thyroid cancers (10–15% of cases), and Hürthle cell carcinomas (3–10% of cases) [1,7,8].

When WDTC is diagnosed, in most situations, a total thyroidectomy (TTX) is performed, followed by the administration of radioactive iodine (I^131^) (RAI), with the intention of inducing a localized radiation treatment leading to complete thyroid ablation [9,10,11]. However, in 15–33% of cases, the malignant thyroid tissue is unable to take up I^131^, leading to an ineffective RAI treatment [12,13]. These patients have either a decreased gene and protein expression level of the sodium iodide symporter (NIS) [14], or NIS has been mis-localized in the intracellular compartment, rather than the cell membrane [15]. This prevents the transportation of RAI into the remaining PTC cells [16,17]. We call this condition RAI-refractory disease (RAIRD), which can be present at diagnosis, but may also develop during the course of a patient’s treatment, making RAIRD an elusive condition to diagnose. As a consequence, additional therapies, such as targeted therapy, chemotherapy, and external beam radiotherapy, will be needed, enhancing the toxicity.

Currently, based on national guidelines, most patients with PTC undergo I^131^ ablation, even though the RAI avidity status of the specific tumor is often unknown [10,11]. However, I^131^ ablation may be accompanied by serious side effects, causing fatigue, salivary and lacrimal gland dysfunction, bone marrow suppression, nausea, a burning sensation in the neck area, and a potential increase of future second primary malignancies [18,19]. Since it is not possible to identify the RAIRD status postoperatively, RAI treatment is usually administered to these RAIRD patients, despite its ineffectiveness, unnecessary side effects, and the delay before receiving alternative therapies [20]. Therefore, there is a clinical need to identify patients with RAIRD early, in order to avoid ineffective I^131^ treatment and prevent an unnecessary delay in receiving alternative treatment strategies, such as radiotherapy.

Patient-derived organoids can be used to study tumor characteristics, as they are known to recapitulate several in vivo aspects, i.e., treatment sensitivity, metastasis potential, genetic alterations, and tumor heterogeneity [21,22,23,24,25]. Two recent studies have shown that patient-derived organoids are able to accurately predict the patient response to treatment [26,27]. Additionally, previous research has shown that most somatic mutations were maintained in lung cancer organoids, as the concordance of somatic mutations ranged from 73% to 100% [28]. Tumor-derived organoids contain putative cancer stem cells (CSC), which possess the ability to self-renew and give rise to multiple different tumor cells. Therefore, in this study, we aimed to develop a PTC organoid model showing the presence of human putative PTC cancer stem cells with the competency to self-renew and form tumor cells and the potential to assess the treatment prediction potential.

## 2. Results

### 2.1. In Vitro Self-Renewal and Organoid Formation from Human PTC and RAIRD Cancer Stem Cells

**Figure 1:**
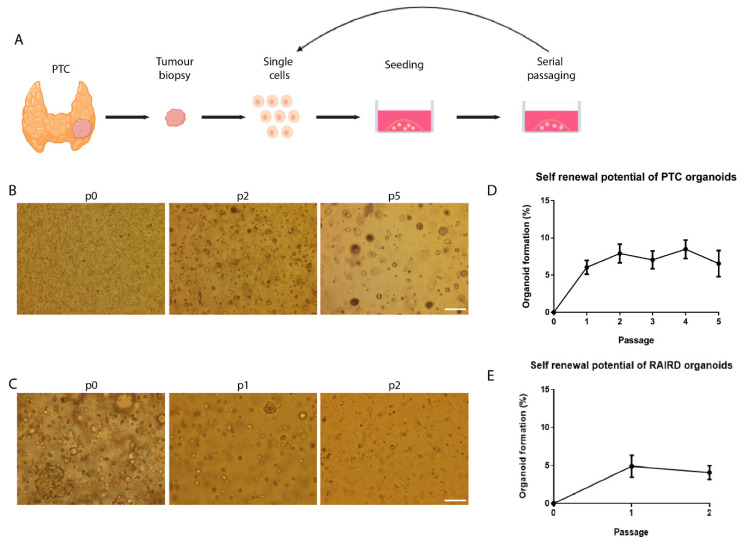
In vitro self-renewal of organoid-forming cells from human putative papillary thyroid cancer (PTC) cancer stem cells. (**A**) Schematic representation of PTC tissue isolation, primary tissue culture, and the self-renewal assay. (**B**) Primary PTC organoids 7 days in culture (p0), and organoids in passage 2 and 5. Scale bar = 250 µm. (**C**) Primary radioactive iodine (RAI)-refractory disease (RAIRD) organoids 7 days in culture (p0), and organoids in passage 1 and 2. Scale bar = 250 µm. (**D**) Self-renewal potential of PTC organoids during passaging (*n* = 13, error bars represent SEM). (**E**) Self-renewal potential of RAIRD organoids during passaging (*n* = 3, error bars represent SEM).

Thirteen PTC biopsies and three RAIRD biopsies were dissociated into single cells and cell clumps using mechanical and enzymatic digestion and cultured in defined medium (Figure 1A). This resulted in the formation of cell clusters (Figure 1B,C, p0). To determine the presence of potential CSC in PTC and RAIRD, the in vitro self-renewal potential was assessed. After the enzymatic dispersion of cell clusters into small clusters composed of 3–4 cells, the ability to form secondary, tertiary, etc. putative PTC or RAIRD organoids in a 3D matrix was assessed. This procedure could be repeated for at least five passages for PTC organoids (Figure 1D), with a stable organoid-forming efficiency (OFE) of about 7%. The RAIRD organoids could be passaged at least three times (Figure 1E), with an OFE of approximately 5%. These results indicate the self-renewal potential of putative PTC and RAIRD organoid-derived cells. This method was previously used in order to determine self-renewal by replating [29], and showed activated stem cell maintenance during long-term self-renewal culture. Furthermore, self-renewal/expansion has been reported for many adult stem cells, including neural [30], intestinal [31, and liver stem cells [32].

### 2.2. Characterization of PTC and RAIRD Organoids

**Figure 2:**
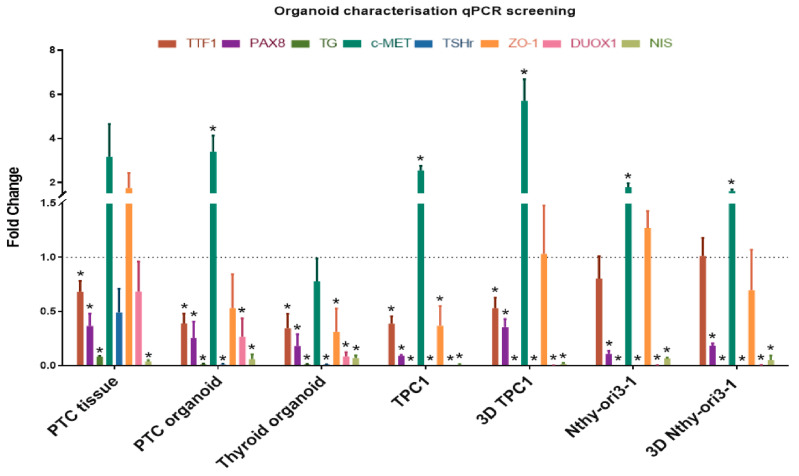
qPCR analysis of the organoid characterization marker panel mRNA level in patient material, organoids, cell lines, and 3D-spheroids (error bars represent the SEM of three biological replicates, normalized to thyroid tissue = dotted line. * *p* < 0.05).

In order to further characterize our PTC and RAIRD organoids, the gene expression of specific thyroid and PTC markers was assessed (Figure 2). We used the normal human thyroid cell line Nthy-ori3-1 and the human thyroid cancer cell line TPC1 [33,34], both cultured in 2D-clones and as 3D-spheroids, as positive controls for marker gene expression. Additionally, we compared normal thyroid tissue, normal thyroid organoids, PTC tissue, PTC organoids, RAIRD tissue, and RAIRD organoids. The panel of genes of interest consisted of thyroid-specific markers (TTF-1, PAX8, TG, and TSHr), a PTC marker (c-MET), and transporters/receptors that are necessary for iodine uptake and thyroid hormone production (DUOX1 and NIS). Thyroid Transcription Factor-1 (TTF-1) and Paired Box Gene 8 (PAX8) are both crucial transcription factors for thyroid organogenesis and differentiation, and are co-expressed solely in the thyroid gland [35]. Thyroid stimulating hormone receptor (TSHr) and thyroglobulin (TG) were included in our panel because stimulation of the TSHr is a crucial step in thyroid hormone synthesis [36], with TG being the precursor to thyroid hormones (T3 and T4) and used as a tumor marker [37]. The proto-oncogene c-MET was included in our panel because of its overexpression in 97% of PTC cases [38]. DUOX1 and NIS were included because of their crucial functions in iodine transportation. The sodium/iodide symporter (NIS) is an integral membrane transporter that mediates active iodine transportation into the thyroid cells, which is then incorporated into TG [39]. For this process, H_2_O_2_ is required, which is produced by Dual oxidase 1 (DUOX1) [40]. Furthermore, we included the marker Zo-1, in order to investigate the presence of tight junctions in our organoid model.

When comparing the gene expression of PTC tissue and PTC organoids, similarities were seen. The expression levels of TTF-1, PAX8, TG, and c-MET were similar between PTC tissue and organoids (Figure 2). However, differences could be seen in the expression levels of Zo-1, DUOX1, and NIS. Moreover, similar differences in Zo-1, DUOX1, and NIS were observed when comparing normal thyroid material with thyroid organoids. This decrease in expression could be a result of the culturing methods yielding partly undifferentiated cells, as previously shown in salivary gland organoids [29]. Additionally, similar expression levels of TTF-1, c-MET, and Zo-1 were discovered when comparing the TPC1 cell line with PTC organoids. However, no expression of TG, TSHr, DUOX1, and NIS was seen in the TPC1 cell line (Figure 2). When the TPC1 cell line was cultured as 3D-spheroids, a more similar expression of TTF1 and PAX8 was discovered, indicating more thyroid differentiation. However, the expression of c-MET and Zo-1 was higher compared to PTC organoids. This indicates that the TPC1 cell line grown in a 2D and 3D condition may be a less suitable model for investigating PTC. When comparing the Nthy-ori3-1 cell line with thyroid organoids, similarities were found in PAX8 and NIS expression. It is known that NIS expression is regulated by the expression of PAX8 [41]. The expression of TTF-1, c-MET, and Zo-1 was increased compared to thyroid organoid levels, and this might be a result of the immortalization of this cell line. No expression of TG, TSHr, and DUOX1 was found when cultured in 2D or 3D conditions (Figure 2). However, a similar expression of PAX8 and NIS was found when comparing 3D-grown Nthy-ori3-1 and thyroid organoids. 

Nevertheless, clear differences were observed when comparing the gene expression of PTC organoids between passages to normal thyroid organoids (Figure S1A). All genes, except for TTF-1, show a reduction of gene expression in passage 2, and an increase in passage 3. In most cases, the expression in passage 3 is increased, compared to normal thyroid organoids, except the NIS expression. This might be an indicator that we are selecting for progenitor and stem cells in passage 2, as seen in other organoids, such as salivary glands [29]. This might show the need for further organoid differentiation protocols for specific experiments.

When comparing the gene expression of RAIRD organoids between passages to PTC organoids, the expression of TSHr and NIS was increased (Figure S1B). The expression levels of PAX8, TG, c-MET, TSHr, and DUOX1 decreased during passaging, resulting in a strong reduction in passage 3, when compared to PTC organoids. As mentioned previously, these markers are essential in differentiated tumor progression, indicating that RAIRD organoids seem to de-differentiate during passaging.

**Figure 3:**
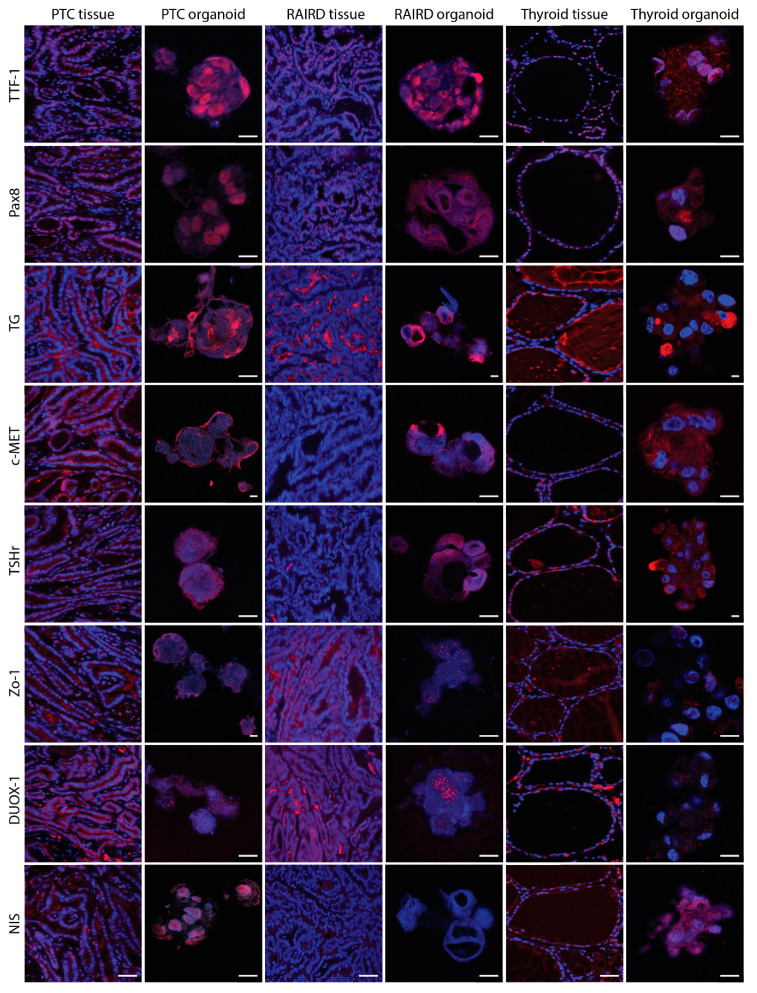
Immunofluorescent characterization of PTC and RAIRD organoids. Representative images of organoids and tissue showing thyroid-specific markers, PTC markers, and markers for thyroid hormone synthesis. Scale bar tissue = 50 µm, and organoids = 10 µm. Markers indicated on the left side are shown as a red fluorescent signal. Nuclei are shown as a blue fluorescent signal.

Next, we used immunofluorescence to detect the protein expression of the same panel of interest (Figure 3). Similar to the gene expression experiments, the 2D- and 3D-grown Nthy-ori3-1 and TPC1 cell lines were used as positive controls (Figure S2). 

When comparing PTC tissue with PTC organoids, similarities were seen in the expression of TTF-1 and PAX8, with mainly nuclear expression in PTC organoids (Figure 3). Additionally, more similarities were found in TG, TSHr, Zo-1, and NIS protein expression. The proto-oncogen c-MET, known to be overexpressed in 97% of PTC cases, is expressed at both a tissue and organoid level. In the latter, the expression of c-MET is located in the membrane, indicative of the active form of c-MET. However, the expression of DUOX1—on a tissue level being expressed in the cytoplasm—seems to form puncta located in the cytoplasm and nucleus of the PTC organoids. It is known that autophagy is a key player in the regulation of DUOX1 localization [42], but generally, DUOX1 is located in the cytoplasm. Therefore, PTC organoids—to a large extent—recapitulate PTC tissue.

**Figure 4:**
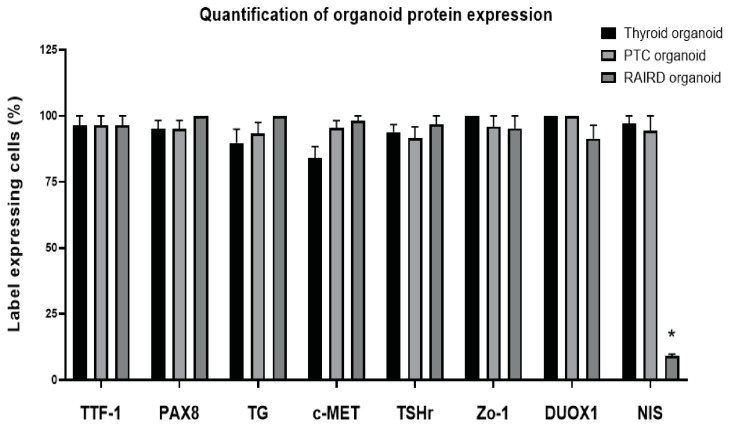
Immunofluorescence quantification analysis of the organoid characterization marker panel (error bars represent the SEM of 10 quantified organoids). Significance is indicated with an asterisk: *p* < 0.001.

A similar protein expression of TTF-1 and PAX8 was noticed when comparing RAIRD tissue with RAIRD organoids. Both markers were located in the nucleus, suggesting active proliferation. However, on a tissue level, no expression of c-MET or TSHr could be detected. In the organoids, both markers were expressed and seen as a diffuse signal throughout the nucleus and partially in the cytoplasm, whereas the expression of both c-MET and TSHr would be expected to be located in the membrane, as in the case of PTC organoids. TG in RAIRD tissue was located in the interstitial space, similar to PTC tissue (Figure 3), whereas the expression in RAIRD organoids was similar to PTC organoids. Puncta formation occurred in the case of Zo-1 and DUOX1 in RAIRD organoids. It has been shown that, for the normal localization of ZO-1 to the plasma membrane, both extracellular calcium and cell–cell contact are necessary [43]. DUOX1 puncta formation was similar to what was noticed in the PTC organoids. Most interestingly, no expression of NIS was detected in RAIRD tissue or organoids (Figure 3, quantified in Figure 4), and this absence was observed in all three patients included in this study (Figure S3). These results indicate that both PTC and RAIRD organoids clearly resemble the tissue of origin.

We detected similarities between thyroid tissue and thyroid organoids in terms of the expression of TTF-1, PAX8, and c-MET. All of these proteins were located in the cytoplasm, indicative of an inactive state, resulting in a non-proliferative state. In the thyroid tissue, TG was detected in the follicular structures, in contrast to the interstitial staining of RAIRD tissue (Figure 3). In the thyroid organoids, TG was seen to be excreted, but also present in the cytoplasm. The expression of TSHr and NIS occurred diffusely throughout the nucleus and cytoplasm, which might be an indication that the transcription and transport of these proteins were activated. DUOX1 expression was again observed in puncta, but less densely grouped in contrast to RAIRD and PTC organoids. We discovered a diffuse Zo-1 expression in thyroid tissue, being located in the follicular structures, but also between cells around these structures. However, in the thyroid organoids, Zo-1 was expressed between some cells, but not abundantly present, as in the case of PTC organoids. It has been shown that Zo-1 plays crucial roles not only in tight junction formation, but also in cell polarization [44]. The pattern that is present in thyroid organoids might be an indication of non-polarized cells. These data indicate that the expression of thyroid markers is clearly seen in all organoids, but also that there is a clear difference between normal and tumor tissue-derived organoids.

For the immunofluorescence of 2D-grown cells, gelatin-coated coverslips were used. This resulted in a background signal in the Cy5 channel. Therefore, we used secondary antibodies with an Alexa 488 label to overcome this background signal (Figure S2). We observed a higher expression of PAX8, c-MET, TSHr, Zo-1, and NIS in the 2D-clones of TPC1 cells when compared to Nthy-ori3-1 cells (Figure S2). When the cell lines were cultured as 3D-spheroids, we found a different pattern. The expression of PAX8, Zo-1, and NIS seemed to be equal, suggesting a higher differentiation state, especially in the Nthy-ori3-1 cells (Figure S2). The expression of TSHr, however, was reduced in the 3D-spheroids of Nthy-ori3-1 cells compared to 3D-spheroids of TPC1 cells. Limited to no expression of DUOX1 in 2D-clones and 3D-spheres of both the Nthy-ori3-1 and TPC1 cells was seen, suggesting a lack of H2O2 production needed for thyroid hormone synthesis. An increased expression, as seen in our immunofluorescence panel for PAX8, TSHr, Zo-1, and NIS, was not detected on a gene expression level of any of the cell lines or organoids, with the exception of c-MET. These data indicate that PTC organoids more closely resemble the PTC tumor tissue when compared to the cell lines. Combining gene expression data and IHC, we observed that our PTC organoids are very similar to PTC tumor tissue, thus making it interesting for use as an in vitro model of putative PTC-derived CSC. Comparing the RAIRD organoids with PTC organoids, we were able to detect a significant difference, with the absence of NIS (quantified in Figure 4). These observed differences might enable the prediction of I^131^ avidity and studies on the mechanism of resistance.

## 3. Discussion

In this paper, we have reported the establishment of a papillary thyroid cancer organoid culture with an organoid forming efficacy of 7%, showing a prolonged self-renewal potential and suggesting the presence of CSC. Furthermore, we have shown that these PTC organoids recapitulate PTC tissue on both gene and protein expression levels. The gene expression levels of TTF-1, PAX8, TG, and c-MET between PTC tissue and organoids are similar. Moreover, we have shown similarities in the protein expression of TTF-1, PAX8, TG, c-MET, TSHr, Zo-1, and NIS. Additionally, similarities were observed between PTC and RAIRD organoids in the expression of TTF-1, PAX8, TG, c-MET, TSHr, and DUOX1. It is highly interesting that the major difference was the complete absence of NIS expression in RAIRD organoids. This striking observation of common and different characteristics of PTC and RAIRD organoids might be indicative of the treatment sensitivity and response of the original tumor.

Previous research has shown that patient conditions are better mimicked when cell lines are cultured as 3D-spheroids, compared to monolayers. This indicates that 3D-spheroids resemble the conditions of the human body better then 2D-cultured cells [45] and indeed, three-dimensional cell cultures are changing the way in which cancers are studied by allowing the non-invasive analysis of patient-derived tissue [46]. Likewise, in this study, we showed that cell lines cultured as 3D-spheroids illustrate a different gene expression pattern more closely related to the tumor tissue when compared to 2D-clones of the same cell line. PTC organoid cultures derived from patient-specific material show an even greater resemblance to PTC tumor tissue, both in terms of gene expression and the protein level. 

Because immortalized cell lines, like the ones used in this study, are unable to represent the tumor heterogeneity, organoids may be used to study patient-specific tumor behavior, and may potentially form a future treatment response prediction tool, as shown in previous research [22,25,26,47,48,49]. Such tumor heterogeneity might be the reason why, in tumor biopsies, RAI uptake is successful. However, when a resistant stem cell population does not take up iodine, the treatment will not be successful. Our methodology makes use of the possibility of culturing putative cancer stem cells (CSC) within organoids, showing the response of the cells that matter and discarding cells that do not make a difference, as these cannot grow under our culture conditions. We used our established PTC organoid culturing method to culture PTC organoids from patients that were RAI avid and patients non-avid for RAI (RAIRD), in order to identify significant differences that might be used as a predictive marker. We were able to culture organoids from known RAIRD tissue with an organoid forming efficacy of 5%, showing clear differences to normal PTC organoids. However, although the recognition of RAIRD has improved over time due to the use of biomarkers such as TG, diagnostic criteria for radioactive iodine avidity are still based on the use of iodine scans and the RAI response and uptake, during or post-treatment. Moreover, the strategies chosen are subject to personal interpretation of the clinical experts. Currently, it is difficult to establish the I131 avidity of a tumor before the administration of a therapeutic dose of I131. A post-therapeutic scan after a high dosage of I131 is the ultimate proof for determining I131 uptake. In high-risk tumors, a “blind” therapy, without proof of uptake and thus efficacy, can be given, with the concomitant possible side effects. In practice, thyroid cancers can behave like poorly differentiated cancers, without I131 uptake, while the pathologist classifies them as well-differentiated. Currently, in the clinical environment, genetic analysis does not help us to conduct further specification. Therefore, the described methodology could help us in the future to subclassify these tumors in advance and determine the RAI avidity, thereby avoiding the unnecessary administration of I131 and preventing side effects.

Currently, there is no well-recognized definition for RAIRD, and no clear guidelines are available for handling patients with RAIRD [50]. These patients are rare and form only a small fraction of the WDTC population, with an estimated incidence of ±5 cases/year/million people [50]. Often, these patients are managed in a non-surgical manner with repeated ineffective RAI treatments, resulting in morbidity such as fatigue, salivary and lacrimal gland dysfunction, a dose-dependent increase of second primary malignancies, bone marrow suppression, nausea, dysgeusia, related diseases, and a burning sensation in the neck area [18,19]. Due to the small population of these patients, obtaining biopsies for organoid cultures is difficult. Since we had very strict selection criteria in our center, we were able to include three patients with RAIRD. These patients did have RAIRD and had been treated with a cumulative dose of 300 mCurie I^131^ without effect, leading to several recurrences, which resulted in surgical resection of the recurrent PTC. 

The limitations of the study include the inter-patient variation and the absence of the original tumor microenvironment. Moreover, we showed a high variation in c-MET and Zo-1 gene expression and the location of protein expression. Both markers are involved in tumor invasion and metastasis, and the overexpression of c-MET has been linked to lymph node metastasis and clinicopathological staging [38]. Additionally, the tight junction marker Zo-1 has been associated with the invasion, development, and progression of several tumors [51,52]. However, in RAIRD organoids, a more diffuse c-MET signal and puncta formation of Zo-1 was seen. This puncta formation might be caused by low calcium conditions. It has been shown that Zo-1 is diffusely located in the cytoplasm under low calcium conditions, but that this observation could be reversed by increasing the calcium levels [43]. Besides the observations of c-MET and Zo-1 expression, we also observed major decreases in gene expression levels of PAX-8, TG, c-MET, TSHr, and DUOX1 in the third passage of RAIRD organoids. These markers are key players in the differentiation of thyrocytes, the production of thyroid hormones, and the release of these hormones. However, on a protein level, none of these decreases were present. Phenotypical analysis in different passages was not possible for the number of organoids that were necessary for self-renewal assessment. Phenotypical analysis of RAIRD organoids in passage 3 would be of interest, because of the dedifferentiation observed (Figure S1b). Nevertheless, a complete absence of NIS expression in all three RAIRD patients was discovered. This decrease might be an indication that these RAIRD organoids are in a less differentiated or even de-differentiated state. Moreover, these observations might be the underlying reason why these patients are I^131^ non-avid. However, the increase in NIS and TSHr gene expression seen in the first passage when comparing PTC organoids and RAIRD organoids might be an indication of an active feedback-loop. This hypothetical feedback-loop might be further activation of the expression of these two genes, and therefore trying to restore iodine uptake, thyroid hormone synthesis, and thyroid hormone release. This increase of gene expression was not accompanied by an increase of protein expression, which might be a result of cellular changes such as the negative regulation of mRNA translation due to the presence of miRNAs, or post-translational modifications [53,54].

Cultured Cultured patient-derived tumor organoids, and expansion by in vitro self-renewal, lack part of the original tumor microenvironment. This microenvironment consists of fluctuating concentrations of growth factors, blood vessels, inflammatory responses, extracellular signals, etc. These signals are crucial factors in cancer therapy research, i.e., anti-angiogenic drugs and immunotherapy [55]. Furthermore, it has been previously shown that microenvironmental factors are able to alter the therapy response of tumor cells, complicating the use of organoids for these studies [47,56]. The effect of the tumor environment on I131 treatment is currently unknown [57] and needs further investigation, such as co-culture with the other components of the tumor microenvironment—studies which are on their way. However, the extremely clear difference in NIS protein expression in RAIRD organoids may indicate predictive potential, even without the tumor environment. Because of the observed absence of NIS in RAIRD organoids cultured form tissue from RAIRD patients, we propose the usage of organoid cultures in the diagnostic process of thyroid cancer. In order to test the patient treatment response before surgical intervention, by obtaining a large needle thyroid biopsy, we would enable the generation of organoids and use these for radioactive iodine refractory screening. Additional research is needed to further explore and confirm the clinical application of this procedure, before providing any basis for clinical decisions. A prospective study of PTC organoids exhibiting a good correlation with later diagnosed RAI avid will show its applicability. 

## 4. Materials and Methods 

### 4.1. Patient Material

Human malignant tissue was obtained from thyroid cancer patients, in the age range of 19–89, with classified papillary thyroid carcinoma (after informed consent and with approval of the ethics committee of the University Medical Center Groningen approval no. 2015/101). The biopsies were transported from the operating room in HBSS supplemented with 1% BSA, on ice, for immediate further processing.

RAIRD tissue was obtained from patients in the age range of 57-83, and these patients were all treated with a cumulative dose of 300 mCurie of I^131^. Additionally, these patients underwent 2–3 thyroid surgeries before acquisition of the material. 

### 4.2. Cell Culture

The human papillary thyroid cancer cell line—TPC1—was kindly provided by Dr. R. Netea-Maier (Radboud UMC, Nijmegen, The Netherlands). The human thyroid cell line Nthy-Ori3-1 was obtained from Sigma-Aldrich (Zwijndrecht, The Netherlands). The cell lines were cultured in RPMI-1640 medium supplemented with 10% fetal calf serum (Thermo Scientific, Paisley, UK) and Pen/Strep (Invitrogen, Bleiswijk, The Netherlands).

### 4.3. Spheroid Culture

The TPC1 and Nthy-ori3-1 cell lines were seeded in an InertGrade 96-well microplate (Brand), at a concentration of 3 × 10^4^ cells/mL. Due to the special InertGrade coating, the cells were unable to attach and clump together, and the spheroids were cultured for 7 days before collection.

### 4.4. Organoid Culture

Obtained biopsies were mechanically digested using the gentleMACS Dissociator (Miltenyi Biotec, Leiden, The Netherlands) and simultaneously subjected to digestion in HBSS (Gibco, NY, USA), 1% BSA (Gibco), 80 mg/mL dispase (Sigma), 1.2 mg/mL collagenase type II (Gibco), and calcium chloride (Sigma) at a final concentration of 6.25 mM for two periods of 15 min in a 37 °C shaking water bath. Digested cells were collected by centrifugation, washed in HBSS/1% BSA solution, and passed through 100 µm cell strainers (BD Biosciences, NJ, USA). The pellet was collected again by centrifugation and resuspended in Dulbecco’s modified Eagle’s medium:F12 medium (DMEM:F12) containing Pen/Strep antibiotics (Invitrogen) and Glutamax (Invitrogen) and adjusted to 1.6 × 10^6^ cells per mL. In total, 25 μL of this cell solution was combined on ice with 50 µL volumes of Basement Membrane Matrigel (BD Biosciences) and deposited in the center of 12-well tissue culture plates. After solidification of the gel in the incubator for 30 min, 1 mL of complete growth medium consisting of 50% conditioned Wnt3a medium; 10% conditioned R-spondin medium (both Wnt3a and R-spondin were collected from producing cell lines); and 40% DMEM:F12 containing B27 (Gibco, 0.5×), HEPES (Gibco, 10 mM), ROCK inhibitor Y-27632 (10 μM, Abcam, Cambridge, UK), Nicotinamide (100 uM, Sigma), Noggin (25 ng/mL, Peprotech, NJ, USA), EGF (20 ng/mL, Sigma), TGF-β inhibitor A 83-01 (5 uM, Tocris bioscience, Bristol, UK), FGF-2 (20 ng/mL, Peprotech), and VEGF-121 (10 ng/mL, Immunotools), was added and replaced on a weekly basis. To test the long-term self-renewing potential, organoids were dissociated and re-plated for the next passage, and this procedure was repeated every three weeks.

### 4.5. Immunofluorescence

For immunofluorescence analysis, the antibodies to TTF-1 (1:100, Abcam), Pax 8 (1:100, Cell marque) for cells/spheroids (1:100, Abcam), TG (1:8000, DAKO), c-MET (1:100, CST), TSHr (1:100, Proteintech, Manchester, UK), Zo-1 (1:300, Proteintech), DUOX1 (1:100, Proteintech) for cells/spheroids (1:50, Proteintech), and NIS (1:100, Proteintech) were used to detect proteins.

TPC1 and Nthy-Ori3-1 cells were plated onto coated glass coverslips and allowed to attach overnight. Attached cells were fixed, permeabilized, and incubated with primary antibody. After incubation with secondary antibodies, the coverslips were mounted using ProLong™ Glass Antifade Mountant containing NucBlue™ Stain (Thermo Fisher).

In the case of the organoids, Matrigel was dissolved by incubation with Dispase enzyme (1 mg/mL for 30 min to 1 h at 37 °C; Sigma). Both spheroids and organoids were washed with PBS/0.2% BSA and centrifuged at 400 *g* for 5 min. The resulting pellet was fixed in 4% paraformaldehyde (15 min, RT) and washed with PBS. Next, the organoids and spheroids were embedded in HistoGel (Richard-Allan Scientific/Thermo scientific) and the gel was subjected to dehydration, followed by embedding in paraffin and sectioning (5 μm). Patient material was fixed in 4% formaldehyde. After dehydration, the tissue was paraffin-embedded and sectioned at a 5 μm thickness. Sections were de-paraffinized and subsequently, Tris-EDTA antigen-retrieval was performed. This was followed by washing, blocking, and incubation with primary antibodies overnight. Slides were then incubated with secondary antibodies, stained with 4′,6-diamidino-2-phenylindole (DAPI), mounted using aqueous mounting medium (DAKO), and imaged using a DM6B microscope (Leica) and a TCS SP8 X confocal microscope (Leica). Ten representative organoids for each marker were imaged at 40× magnification. The percentage of label-presenting cells was obtained by dividing the number of label-presenting cells by the total number of nuclei imaged. Results were presented as the mean percentage of label-presenting cells, quantified from ten organoids.

### 4.6. Cell RNA Extraction and qRT-PCR 

Total RNA from cell lines, spheroids, organoids, and patient material was prepared (RNeasy™ Mini Kit, Qiagen), following the manufacturer’s instructions. In total, 500 ng total RNA was reverse transcribed by using 1 µL 10 mM dNTP Mix, 100 ng random primers, 5× First-strand Buffer, 0.1 M DTT, 40 units of RNase OUT, and 200 units of M-MLV RT, with a total of 20 µL for each reaction. A quantitative polymerase chain reaction (Bio-Rad, Veenendaal, The Netherlands)(qPCR) was performed using Bio-Rad iQ SYBR Green Supermix, according to the manufacturer’s instructions. A total of 100 ng cDNA was mixed with PCR buffer, SyberGreen, and both forward and reverse primers for genes of interest, with a total volume of 13 µL for each sample. A three-step PCR reaction was applied subsequently. All agents mentioned above were obtained from Invitrogen. Oligo sequences of primers used were as follows: TTF-1 fwd, 5′-ATGTACCGGGACGACTTGGAA-3′; TTF-1 rev, 5′-CAATGCCTGTCAGGGCTAGAA-3′; PAX8 fwd, 5′-AAGTGCAGCAACCATTCAACC-3′; PAX8 rev, 5′-CTGCTCTGTGAGTCAATGCTTA-3′; TG fwd, 5′-AAGCCTCTGCAATGTGCTC-3′; TG rev, 5′-GGACATAGCCTGGGCTGAC-3′; c-MET fwd, 5′-CAGATGTGTGGTCCTTTG-3′; c-MET rev, 5′-ATTCGGGTTGTAGGAGTCT-3′; TSHr fwd, 5′-AAAGAGCTCCCCCTCCTAAA-3′; TSHr rev, 5′-TTGGTCAGGTCAGGGAACAT-3′; Zo-1 fwd, 5′-CGGTCCTCTGAGCCTGTAAG-3′; Zo-1 rev, 5′-GGATCTACATGCGACGACAA-3′; DUOX1 fwd, 5′-CCAGCAATCATCTATGGGGGC-3′; DUOX1 rev, 5′-TGGGGCCGCTGGAACC-3′; NIS fwd, 5′-TTCCTCTGGATGTGCCTGGGC-3′; NIS rev, 5′-GTACTCGTAGGTGCTGGTGAG-3′; YWHAZ fwd, 5′-GATCCCCAATGCTTCACAAG-3′; YWHAZ rev, 5′-TGCTTGTTGTGACTGATCGAC-3′.

## 5. Conclusions

The results presented in this paper provide the first evidence that patient-derived PTC organoids recapitulate PTC tissue. Organoids cultured from patient-derived material showed a self-renewal potential for several passages over numerous weeks, although the RAIRD organoids were limited in self-renewal potential, possibly due to signs of de-differentiation. We have shown the first indications that NIS expression screening might be an option for gaining more knowledge on the I^131^ avidity in patients before surgical intervention and might be valuable in determining whether a patient should be treated with I^131^ or would be a better candidate for targeted therapy, chemotherapy, external beam radiotherapy, or no direct postoperative therapy at all. 

The ability to culture PTC as organoids over long periods of time represents a platform for the further study of PTC and its response to cancer treatment is a good start. The results of our work provide the first evidence that patient-derived PTC organoids recapitulate PTC tissue. Moreover, our results indicate that patient-derived organoids might be a suitable tool for the identification of patients with RAIRD. Furthermore, the current study opens new avenues for the development of new therapies and allowing early non-responding patient identification to optimize further treatment strategies.

## Supplementary Materials

The following are available online at https://www.mdpi.com/2072-6694/12/11/3212/s1: Figure S1: Changes in gene expression between passages in PTC and RAIRD organoids; Figure S2: Immunofluorescent analysis of proteins of interest in 2D- and 3D-grown Nthy-ori3-1 and TPC1 cell lines; Figure S3: Immunofluorescent analysis of NIS expression in RAIRD organoids from patients four and five.
