# Supplementary material for: Patient-Derived Papillary Thyroid Cancer Organoids for Radioactive Iodine Refractory Screening
Source: Cancers (Basel). 2020 Oct 31;12(11):3212. doi: 10.3390/cancers12113212 (PMC7692469; doi:10.3390/cancers12113212)
Supplement: Supplementary file 1 [file cancers-12-03212-s001.pdf]

cancers

Supplementary Materials: Patient-Derived Papillary Thyroid Cancer Organoids for Radioactive Iodine Refractory Screening

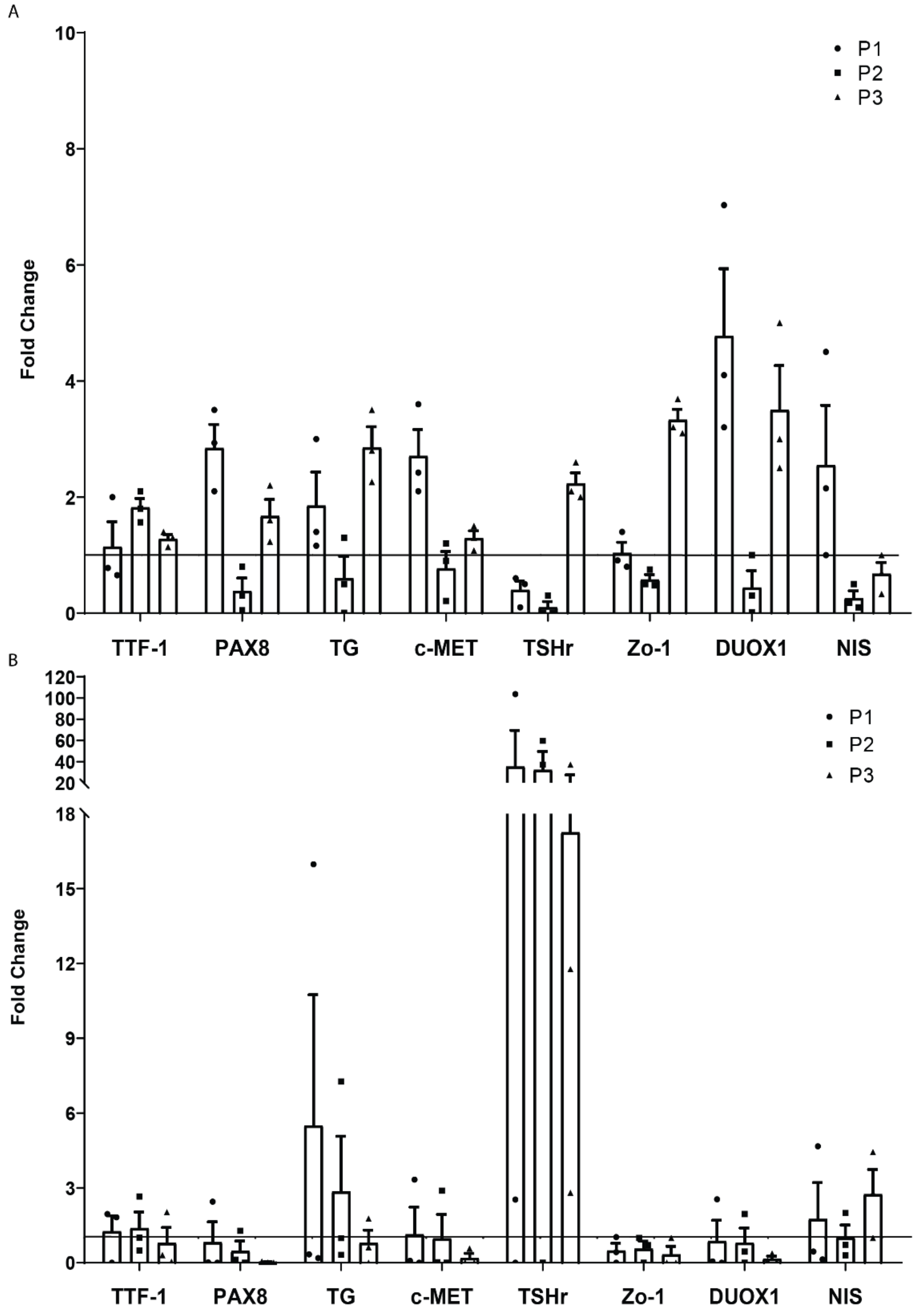

Figure S1. Changes in gene expression between passages in PTC and RAIRD organoids.

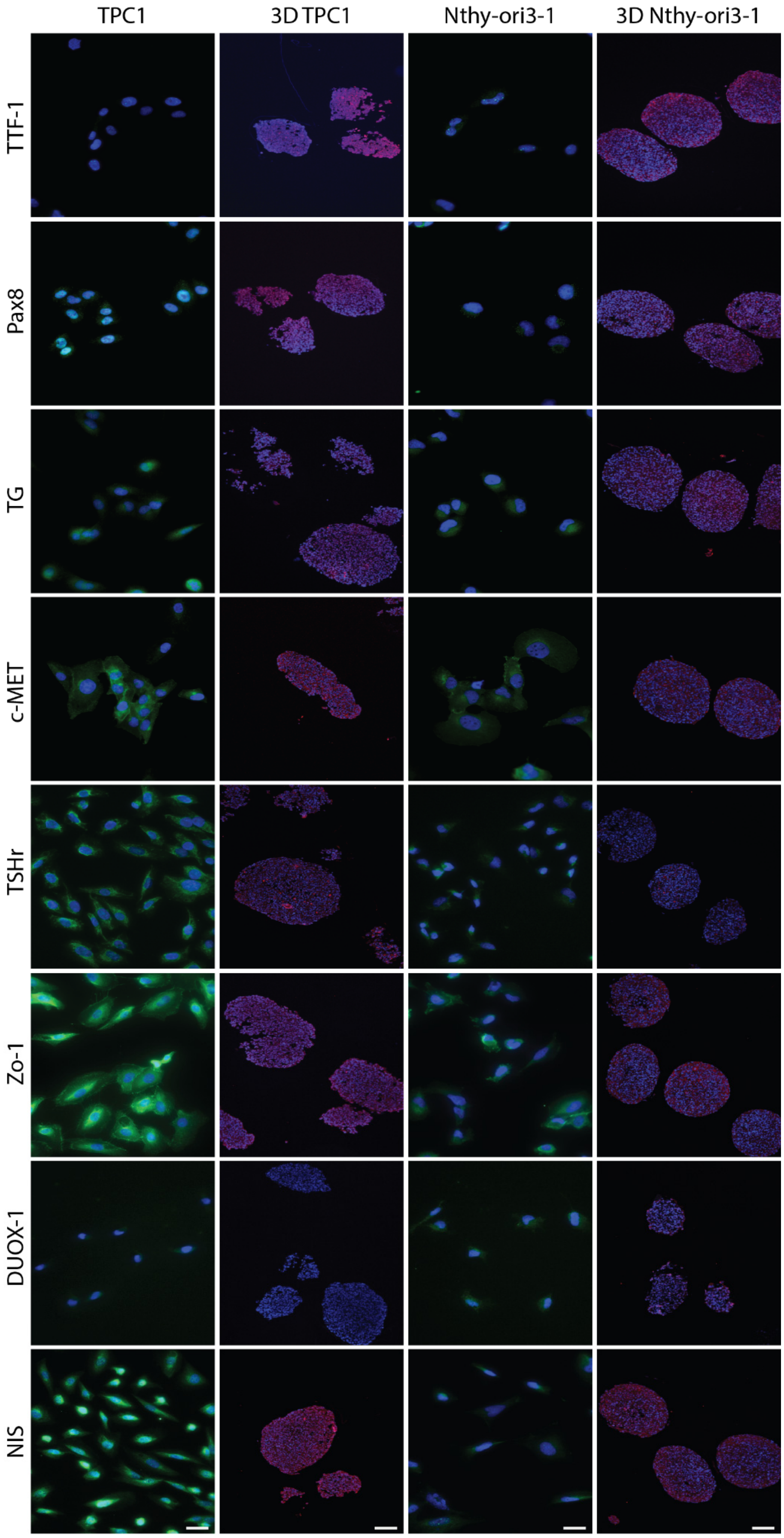

Figure S2. Immunofluorescent analysis of proteins of interest in 2D- and 3D-grown Nthy-ori3-1 and TPC1 cell lines.

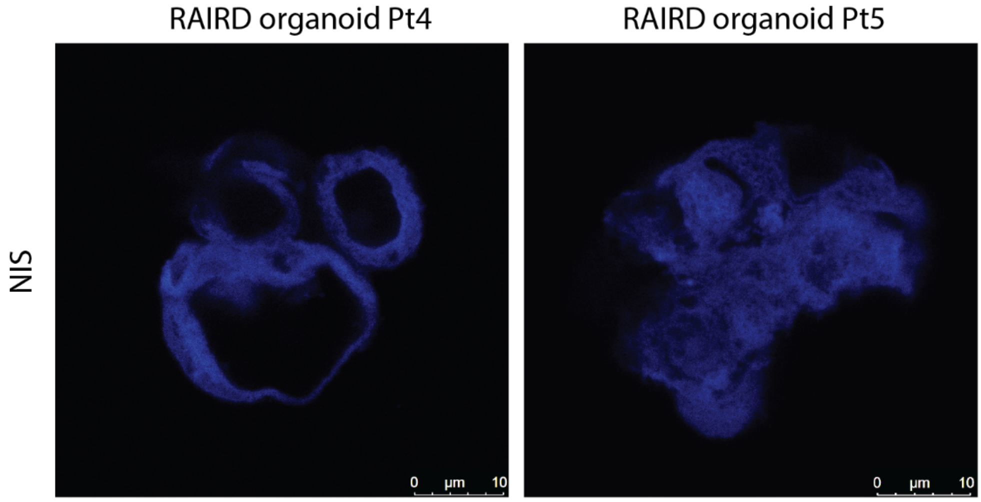

Figure S3. Immunofluorescent analysis of NIS expression in RAIRD organoids from patients four and five.

© 2020 by the authors. Licensee MDPI, Basel, Switzerland. This article is an open access article distributed under the terms and conditions of the Creative Commons Attribution (CC BY) license (http://creativecommons.org/licenses/by/4.0/).
